# Coronavirus-disease-2019-induced antiphospholipid-like syndrome: a case report

**DOI:** 10.1186/s13256-021-02966-4

**Published:** 2021-07-29

**Authors:** Fatemeh Bahramnezhad, Banafsheh Ghorbani, Meysam Ghaedrahamt, Hamidreza Jamaati

**Affiliations:** 1 Nursing and Midwifery Care Research Center, School of Nursing and Midwifery, Tehran University of Medical Sciences, Tehran, Iran; 2 Spiritual Health Group, Research Center of Quran, Hadith and Medicine, Tehran University of Medical Sciences, Tehran, Iran; 3 Student Research Committee, School of Nursing and Midwifery, Hamadan University of Medical Sciences, Hamadan, Iran; 4 Student Research Committee, School of Nursing and Midwifery, Shahid Beheshti University of Medical Sciences, Tehran, Iran; 5 Chronic Respiratory Diseases Research Center, NRITLD, Shahid Beheshti University of Medical Sciences, Tehran, Iran

**Keywords:** COVID-19, Antiphospholipid syndrome, Antiphospholipid, Antibodies

## Abstract

**Background:**

This paper describes a case of antiphospholipid syndrome-like condition caused by coronavirus disease 2019. The medical community still faces many diagnostic and therapeutic challenges vis-à-vis coronavirus disease 2019. Ultimately, coronavirus disease 2019 is diagnosed on the basis of laboratory and radiological findings. Considering the high rate of mortality due to coagulation abnormalities and thrombosis among coronavirus disease 2019 patients, it is important to pay attention to the differential diagnoses of coronavirus disease 2019 and other diseases following thrombotic events.

**Case description:**

The patient was a 56-year-old Iranian man who underwent coronary artery bypass graft surgery and mitral valve repair. During hospitalization, the patient showed an elevated level of anticardiolipin antibody (immunoglobulin G isotype), antiphospholipid antibodies, and thrombosis in the brachial artery of the left hand, based on which a differential diagnosis of antiphospholipid syndrome was made. However, ultimately, the coronavirus disease 2019 polymerase chain reaction test and computed tomography scan of the lungs showed that the patient had coronavirus disease 2019.

**Conclusion:**

According to the few studies performed on coronavirus disease 2019 patients, elevated levels of the isotypes of antiphospholipid antibodies in coronavirus disease 2019 patients create conditions similar to antiphospholipid syndrome, which, in the absence of reliable coronavirus disease 2019 testing, can lead to misdiagnosis and consequently delayed or improper treatment. Therefore, to provide timely and appropriate treatment, it is important to pay attention to differential diagnosis.

## Introduction

Since the emergence of the first cases of coronavirus with acute respiratory distress syndrome, physicians of the affected countries have proposed various definitions and treatment methods and different classifications of the disease on this basis. However, coronavirus disease 2019 (COVID-19) differs from the clinical manifestations of acute respiratory distress syndrome (ARDS) in that it can affect the vascular endothelium, cause thrombosis, and progress to multiple organ failure [1]. The mortality rate of this infectious disease is reported to be 3.7% [2]. Multiple therapeutic, supportive, pharmacological, and mechanical approaches have been recommended for infected patients [3], which include extracorporeal membrane oxygenation [4], medication with interferon beta and ribavirin [4, 5] and remdesivir [6], and corticosteroid therapy [7]. One way to diagnose COVID-19 is to search for elevated levels of immunoglobulin G (IgG) and IgM antibody response to severe acute respiratory syndrome coronavirus 2 (SARS-CoV-2), which is greatly beneficial if used in conjugation with polymerase chain reaction (PCR) test to detect false negatives [8]. Antiphospholipid syndrome (APS) is an autoimmune disorder characterized by thrombosis as its main pathological process and symptoms such as arterial and venous thrombosis, recurrent miscarriage in pregnant women, thrombocytopenia, and neurological and cardiac disorders [9]. The diagnosis criteria for APS are based on the detection of abnormal levels of at least one of the most common antiphospholipid antibodies, viz. lupus anticoagulant (LA), anticardiolipin antibodies (IgM and IgG isotype in medium-to-high titer), or anti-beta 2 glycoprotein I [anti-β2GPI] antibodies (IgM and IgG isotype), along with thrombotic events in arterial or venous blood flow, thrombosis in tissues and organs, or pregnancy complications. It has been recommended to reexamine the patient at least two times in 12 weeks to confirm this diagnosis [9, 10]. In view of the continuation of the COVID-19 pandemic and the diagnostic and therapeutic challenges that we still face vis-à-vis this disease, this paper reports on a patient who had clinical symptoms of COVID-19 but received a differential diagnosis of APS in an open heart intensive care unit.

## Case presentation

**Fig. 1:**
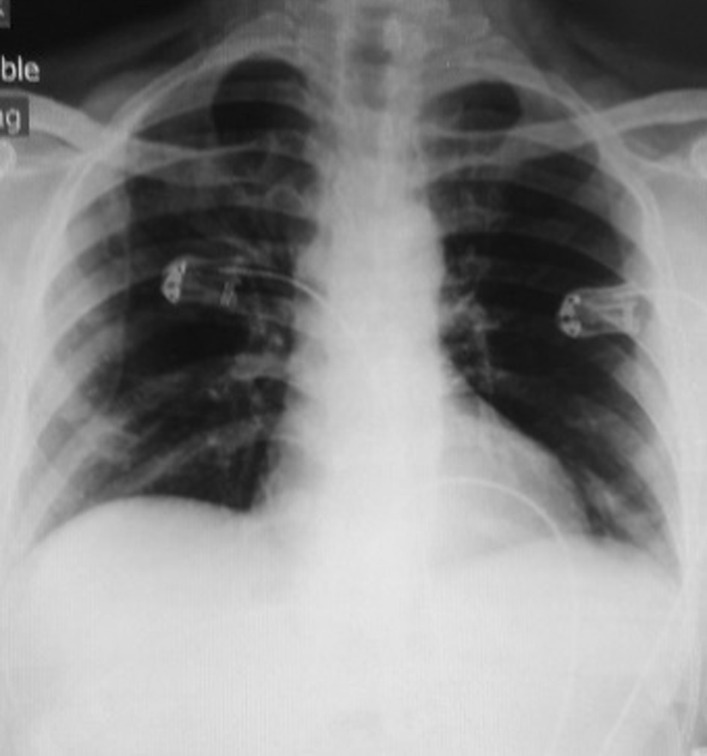
Initial chest X-ray, anterior–posterior view

The patient was a 56-year-old Iranian man with a history of high blood pressure, benign prostate hyperplasia, and hypothyroidism with a diagnosis of three-vessel disease and mitral valve stenosis who was admitted to Medical Center A on 10 July 2020 for coronary artery bypass graft (CABG) and mitral valve repair and underwent surgery on 14 July 2020. In surgery, three grafts were performed on the vessels of the left anterior descending (LAD), obtuse marginal (OM), and left circumflex (LCX), and the mitral was repaired. In the initial preoperative examination, the patient was checked for COVID-19, but the chest X-ray of the lung was normal (Fig. 1), and the result of the COVID-19 RT-PCR test was negative.

**Fig. 2:**
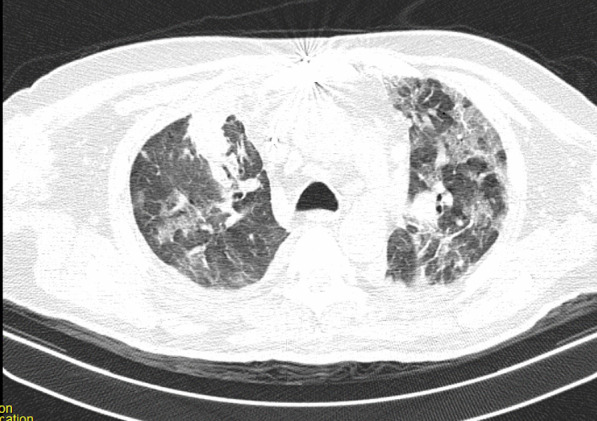
Chest CT recommended considering infection with COVID-19

**Fig. 3:**
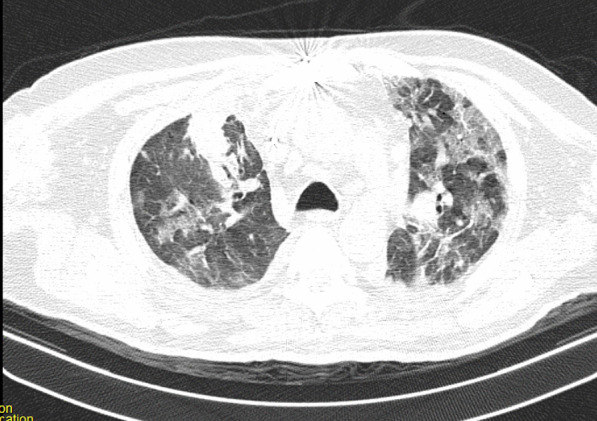
Chest CT recommended considering infection with COVID-19

After the operation, the patient could not be weaned from the ventilator because of poor arterial blood gas (ABG) and peripheral oxygen saturation (SpO_2_) (respiratory acidosis and drop in SpO_2_). The pulmonologist ordered a chest X-ray and CT scan of the lungs on the seventh day of hospitalization. The CT showed several ground-glass lesions in the left and right lungs, based on which the radiologist recommended considering infection with COVID-19 (Fig. 2). The chest X-ray also showed opacity in the alveoli at the base of the lung (Fig. 3).

The patient underwent the COVID-19 PCR test twice on day 5 and day 11 of hospitalization, both of which returned negative. Three days after the operation, the patient’s pulmonary condition improved, and he met the criteria for weaning from the ventilator. At that point, bi-level positive airway pressure (BiPAP) therapy was also prescribed to control the lung problems, reduce CO_2_ levels, and increase SpO_2_, and the therapy was successful in improving the patient’s lung condition. In the meantime, the patient started to experience paresis in the left hand, acrocyanosis in fingertips, and coldness and numbness in other parts of the left hand. On examination, the brachial pulse was not palpable. After performing arterial and venous color Doppler ultrasound, the radiologist reported signs of thrombosis in the brachial artery. There were also signs of lack of proper blood flow in the radial and ulnar arteries. Based on these observations, the patient became a candidate for brachial artery embolectomy. Brachial artery embolectomy was performed, and the sensation, movement, and pulse of the hand normalized. Table 1 presents the patients’ test results during hospitalization, which indicated leukopenia, thrombocytopenia, and increased inflammatory markers.

**Table 1:** Patient’s test results during hospitalization

Day of hospitalization	PLT	WBC	PT	PTT	INR	ESR	CRP	PCR	D-dimer
Day 2	255	6.850	15.5	47	1.35	12	4	Negative	–
Day 6	220	6.900	15	46	1.22	10	3	–	–
Day 8	205	4.855	12	90	2	–	–	Negative	0.3
Day 9								Negative	
Day 11	90	4.200	35	102	3.10	–	–	–	–
Day 12	108	3.450	31.5	105	3.3	–	–	–	–
Day 13	111	3.900	46.1	105.5	3.5	–	–	–	–
Day 15	184	3.232	44.42	112.4	3.8	117	160.4	Positive	3.51

(−): not tested

*PLT* platelet, *WBC* white blood cell, *PT* prothrombin time, *PTT* partial thromboplastin time, *INR* international normalized ratio, *ESR* erythrocyte sedimentation rate, *CRP* C-reactive protein

Due to the possible heparin-induced thrombocytopenia, According to the hematologist, heparin drip was discontinued and argatroban drip was started at a dose of 2 μg/kg/minute with daily PTT check. Considering platelet depletion despite receiving argatroban, clinical signs of thrombosis in the left hand, and positive test result for antiphospholipid antibodies, on day 12 of hospitalization, a differential diagnosis of APS was made, and plasmapheresis was prescribed accordingly (2.5 liters of plasma for at least 5 days). The results of the tests of antibodies are presented in Table 2.

**Table 2:** Results of the tests of autoimmune and antiphospholipid antibodies

Date (MM/DD/YY)	dsDNA (anti-dsDNA)	Anticardiolipin antibodies (IgG)	Anticardiolipin antibodies (IgM)	Antiphospholipid antibodies(IgG)	Antiphospholipid antibodies(IgM)
Day 12	(−)0.73	(+)280	(−)12.5	(+)230	(−)10.3
Day 24	(−)0.74	(−)16	(−)13	(−)12	(−)11

**Fig. 4:**
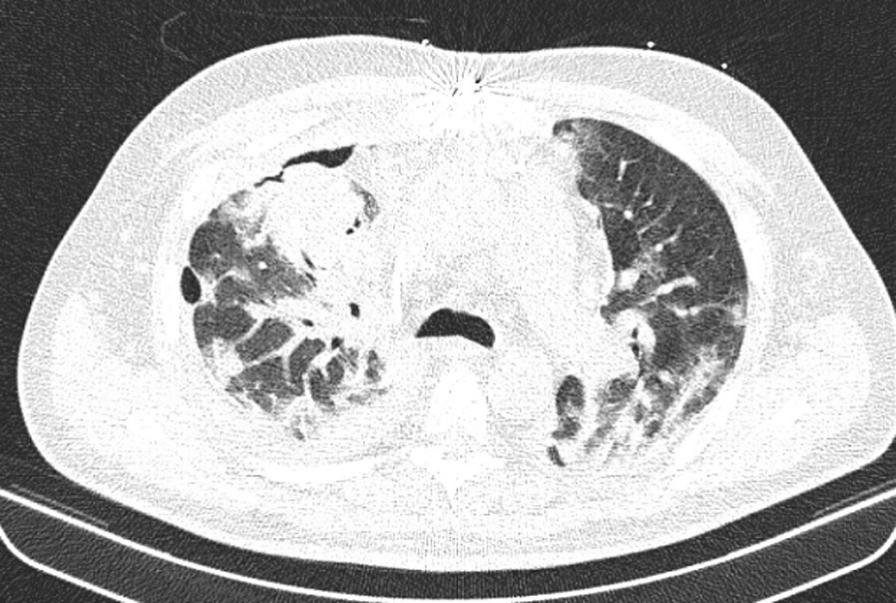
CT image of the lungs, axillary view, showing bilateral opacity

At the same time, the patient was also checked for other differential diagnoses, namely cardiovascular disease (CVD) and vasculitis, which were ruled out. Therefore, the plasmapheresis treatment was started on 21 July 2020. The patient showed improvement after undergoing five cycles of plasmapheresis in 1 week. However, the patient’s third COVID-19 PCR test returned positive, and the subsequent CT scan of the lungs showed signs of COVID-19 infection. Ultimately, a definitive diagnosis of COVID-19 was made based on additional tests conducted in the next period (Fig. 4).

**Fig. 5:**
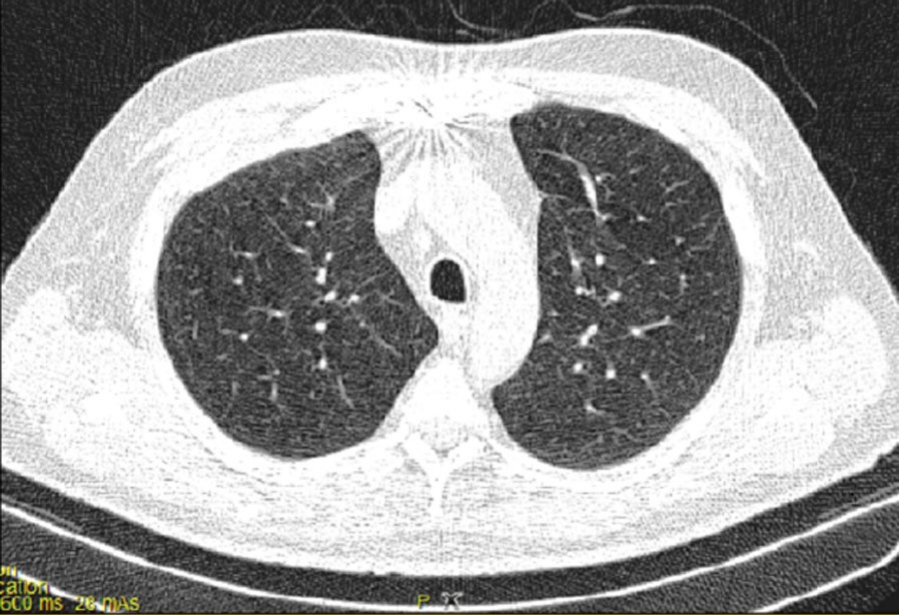
CT image of the lungs, axillary view, showing recovery after treatment

The patient was given dexamethasone 8 mg three times daily (intravascular), hydroxychloroquine tablets 200 mg twice daily, remdesivir injection 200 mg on day 1 and 100 mg from day 2 to day 5, and interferon-beta 250 mg every 48 hours (subcutaneous). On the seventh day since starting COVID-19 treatment, CT scan of the lungs showed that the patient was recovering from COVID-19 (Fig. 5), and the final PCR test result returned negative. Finally, the patient was discharged from the hospital with relatively good general condition, normal lung function without respiratory distress, SpO_2 _ 92%, and without needing supplemental oxygen.

## Discussion

Common coagulation-related observations in COVID-19 patients include increased D-dimer level, decreased platelet count, increased interleukin level, abnormal tumor necrosis factor, increased lactate dehydrogenase, and prolonged prothrombin time (PT) [11]. The patient of this report also showed increased D-dimer and PT and decreased platelet count [11, 12]. However, another differential diagnosis for this patient was APS. This disease usually targets phospholipid proteins, and the persistent abnormal presence of these antibodies over a period of at least 3 months is essential for the diagnosis of APS [13]. One of the diagnostic criteria for this syndrome is the presence of one or more clinical manifestations such as vascular thrombosis, platelet depletion, and heart valve disease, followed by observing abnormal levels of antiphospholipid antibodies over a period of time [14]. However, patients with acute diseases or infections like COVID-19 may also temporarily show high levels of these antibodies [15]. Upon detecting signs of thrombosis, we first tested the patient for antiphospholipid antibodies, and as presented in Table 2, this test returned positive. However, COVID-19 patients may also show elevated levels of some isotypes of antiphospholipid antibodies, which may follow a fluctuating course according to several studies. In some patients, antiphospholipid antibodies may disappear within a few weeks. In our patient, the antibody titer decreased and returned to the normal range over the next 15 days, which ruled out the diagnosis of APS. Therefore, COVID-19 patients with positive antiphospholipid antibodies (COVID-19-induced APS-like syndrome) should be monitored for at least 12 weeks (tested twice or more for antiphospholipid antibodies within 12 weeks) to confirm or reject the differential diagnosis of APS and provide appropriate treatment if necessary [13]. By binding to the surface of monocytes, platelets, and endothelium, antiphospholipid antibodies can also cause higher expression of tissue factor and the release of clotting microparticles [16]. As explained in the biography, thrombotic events in the brachial artery are indicative of this process. Therefore, it is recommended to use heparin or low-molecular-weight heparin (such as enoxaparin) to prevent thrombotic events following COVID-19 in patients who are not contraindicated [15]. Plasma exchange can also help treat thrombotic microangiopathy and eliminate additional inflammatory mediators. In our case, the patient underwent plasmapheresis, which, as mentioned, reduced the level of antibodies and set him on the path to recovery. Another important point is to pay attention to the patient’s test results during hospitalization. Our patient showed increased erythrocyte sedimentation rate (ESR) and CRP, thrombocytopenia, and leukopenia after surgery. As research has shown, the laboratory findings that indicate COVID-19 are usually nonspecific and do not follow the same pattern in all patients. However, most studies agree that people infected with COVID-19 are likely to show increased levels of ESR and CRP, thrombocytopenia, and leukopenia [17, 18], as was the case in our patient. Another important issue is the loss of time that could be spent on providing more effective treatment because of incorrect sampling for the COVID-19 PCR test. As explained, in the reported case, a chest X-ray indicated the presence of COVID-19, but the PCR test returned negative twice, leading to a delay in the treatment process.

## Conclusion

Elevated levels of the isotypes of antiphospholipid antibodies in COVID-19 patients create conditions similar to antiphospholipid syndrome, which, in the absence of reliable COVID-19 testing, can lead to misdiagnosis and consequently delayed or improper treatment). Therefore, in areas where the COVID-19 epidemic is still present, patients with signs of antiphospholipid syndrome should undergo an at least 12-week long monitoring of inflammatory factors, thrombotic events, and antiphospholipid antibodies in order to differentiate antiphospholipid syndrome from COVID-19-induced thrombosis.

## Data Availability

Not applicable.

## Abbreviations

BiPAP: Bi-level positive airway pressure
